# The Use of the Target Trial Approach in Perinatal Pharmacoepidemiology: A Scoping Review Protocol

**DOI:** 10.3389/fphar.2022.904824

**Published:** 2022-07-22

**Authors:** Lisiane Freitas Leal, Sonia Marzia Grandi, Daniel Marques Mota, Paulo José Gonçalves Ferreira, Genevieve Gore, Robert William Platt

**Affiliations:** ^1^ Faculty of Medicine and Health Sciences, Department of Epidemiology, Biostatistics and Occupational Health, McGill University, Montreal, QC, Canada; ^2^ Child Health Evaluative Sciences, The Hospital for Sick Children, Toronto, ON, Canada; ^3^ Division of Epidemiology, Dalla Lana School of Public Health, University of Toronto, Toronto, ON, Canada; ^4^ Brazilian Health Regulatory Agency (Anvisa), Brasília, Brazil; ^5^ Schulich Library of Physical Sciences, Life Sciences and Engineering, McGill University, Montreal, QC, Canada

**Keywords:** scoping review, target trial emulation, preconception, perinatal, postpartum, pharmacoepidemiogy

## Abstract

**Background:** Pregnant and postpartum women have been historically excluded from clinical trials, with data on the safety of drugs relying on observational research. Methodological concerns regarding the timing and dosing of medications, data sources, study designs, and methods used for estimating associations are still problematic in observational studies. Answering causal questions is even more complex. Despite the increased interest in emulating target trials using observational data, little is known about this approach in perinatal pharmacoepidemiology.

**Objective:** This scoping review protocol aims to describe the methodology for assessing the available literature concerning emulating target trials for studying outcomes in women exposed to medications in the preconception, perinatal, or postpartum periods.

**Methods and Analysis:** We will follow the methods detailed in the Joanna Briggs Institute reviewer’s manual. We will adopt the six-stage framework recommended by Arksey and O'Malley and Levac and others. Web scraping techniques will be used for identifying relevant studies. Two authors will select articles based on the title and abstract, with discrepancies resolved by consensus, by a third reviewer. Preferred Reporting Items for Systematic reviews and Meta-Analyses extension for Scoping Reviews flow diagram will be presented to reflect the search process. We will use existing statements to identify quality gaps in the current literature. Variables related to the content for perinatal pharmacoepidemiologic research will be included. The Risk Of Bias In Non-randomised Studies - of Interventions (ROBINS-I) will guide the assessment of the target trial emulation (i.e., treatment strategies compared, assignment procedures, follow-up period, outcome, and causal contrasts).

**Discussion:** Data regarding the safety of drugs taken, prior to and during pregnancy and while lactating are lacking and it is necessary to understand how we can answer these questions using rigorous methods in observational research. Through this scoping review, we intend to understand to what extent the target trial approach is being used in perinatal pharmacoepidemiology and provide recommendations to improve its use in this field.

**Ethics and Dissemination:** Secondary data from published scientific articles will be used, not requiring approval by the Research Ethics Committee with human beings. Findings will be submitted to a peer-reviewed journal.

## Background

Research in women’s health is usually focused on specific periods of a women’s life such as the reproductive or perinatal period (World Health Organization, 2009). Pregnancy and childbirth are normal physiological and social processes that carry health risks and require follow-up and care. Pregnancy may unmask or worsen a pre-existing condition (Torgersen and Curran, 2006), leading to interventions to reduce the burden and risk of long-term morbidity (Neiger, 2017). Although pharmacotherapy is typically the first-line therapy, questions relating to the effectiveness and safety of medications used in pregnancy and lactation, still remain.

The physiological changes that occur during pregnancy affect both the pharmacokinetics of drugs (i.e., absorption, distribution, metabolism, and elimination) and the pharmacodynamics, influencing the mechanism of action and magnitude of observed pharmacological effects (Zhao et al., 2014). Other factors might also contribute to the change in pharmacokinetics/pharmacodynamics during pregnancy including pre-existing comorbidities, maternal age, race, ethnicity, body weight, singleton vs. multiple gestations, gestational age, smoking history, alcohol usage, dietary habits, and illegal drug use, as well as other behavioral changes (Moya et al., 2014; Zhao et al., 2014). Teratogenic effects are the leading concern for medication use prior to and during pregnancy; while their use following delivery and while lactating raises concerns for the health of neonates and the long-term development of the child (Wecker et al., 2019). Given the lack of data on use of medications during pregnancy, little guidance exists for practitioners to determine whether the benefit outweighs the risks of pharmacological treatments (Ansari et al., 2016). Pregnant women have been historically excluded from clinical trials (Food and Drug Administration, 2018; van der Graaf et al., 2018; Yakerson, 2019; Bianchi et al., 2021), with data on the safety of newly marketed drugs relying on post-marketing surveillance (Huybrechts et al., 2021). There is, therefore, a need for rigorous observational research, to elucidate the effects of drugs in this population.

Nevertheless, evaluating the effectiveness and safety of medications during pregnancy presents major challenges. Methodological concerns regarding the timing and dosing of medications, data sources, study designs, and methods used for estimating associations are still problematic in observational studies (Horton et al., 2019; Huybrechts et al., 2019). Answering causal questions is even more complex. More recently, Hernan and others (Hernán and Robins, 2016) have proposed a framework for conducting comparative effectiveness research. More specifically, they proposed the use of large databases to emulate hypothetical pragmatic randomized trials, also called target trial emulation (Hernán and Robins, 2016).

The use of observational studies analyzed like randomized experiments was first demonstrated in 2008 (Hernán et al., 2008). Briefly, this method advocates the adoption of seven key components that should be clearly outlined in a target trial protocol: the eligibility criteria, treatment strategies being compared (including their start and end times), assignment procedures, follow-up period, outcome of interest, causal contrast(s) of interest, and analysis plan (Hernán and Robins, 2016). Despite the increased interest in emulating target trials using observational data, gaps have been identified, such as the misalignment of start of follow-up, eligibility, and treatment assignment, as well as the lack of complete knowledge on confounders (Hernán et al., 2016). Additionally, little is known about use of target trial emulation in perinatal pharmacoepidemiology research, in which key elements including, exposure ascertainment and etiologically relevant time of exposure are fundamental for interpreting outcomes (Fell et al., 2021).

Two recent articles have adopted the target trial approach to estimate the comparative effectiveness and safety of treatments before conception (Caniglia et al., 2018; Yland et al., 2022). However, the use of this method is likely more readily adopted by pharmacoepidemiologists working in perinatal research. Through a scoping review, we aim to understand the use of the target trial approach to answering causal questions in perinatal pharmacoepidemiology.

## Scoping Review Aims

The aim of this protocol is to describe the methodology for assessing the available literature concerning emulating a target trial for studying outcomes in women exposed to medications in the preconception, perinatal, or postpartum periods. The specific objectives will be *i*) to describe the identified studies by type, year, and country *ii*) to describe the inclusion of the components of the target trial protocol of included studies *iii*) to identify knowledge gaps (i.e., reporting of the components of a target trial protocol, data sources availability, quality, research located in few centers etc.), and, *iv*) to provide recommendations for future research relating to the target trial approach.

## Methods and Analysis

The proposed scoping review will follow the methods detailed in the Joanna Briggs Institute (JBI) reviewer’s manual (Peters et al., 2020). We will adopt the six stage framework recommended by Arksey and O'Malley (Arksey and O'Malley, 2005) and Levac and others (Levac et al., 2010).

### Stage 1: Identifying the Research Question

The Population, Concept and Context (PCC) elements (Peters et al., 2020) will guide the title, objective of the scoping review, question, and inclusion criteria. The question for the scoping review is: What is known from the existing literature about emulation of randomised trials using observational data (i.e., target trial emulation) for assessing outcomes related to medication exposure before, during or after pregnancy (post-delivery and during lactation)?

### Stage 2: Identifying Relevant Studies

A three-step search strategy for identifying relevant studies is recommended according to JBI (Peters et al., 2020): *i)* searching online databases, followed by an analysis of the text words contained in the title and abstract of records identified as relevant, and of the index terms used to describe the articles; *ii)* using all identified keywords and index terms across all included databases; *iii)* searching the reference list of all identified reports and articles for additional studies.

For our study, the first two steps will be automated using Web scraping techniques (Najork et al., 2018), data cleaning and deduplication (Ganti et al., 2018; Kaushik et al., 2018) developed by our group using the Python programming language (Python Software Foundation, https://www.python.org/) and previously used for various types of reviews, including scoping reviews (Mota et al., 2020). Briefly, Web scraping is a technique for extracting Web contents. Through this process a software agent, also known as a Web robot (scraper), mimics the browsing interaction between human and Web servers in a conventional Web navigation, extracting and combining contents of interest from the Web in a systematic way (Glez-Peña et al., 2013). The structure and content of a Web page are encoded in Hypertext Markup Language (HTML). A scraper is built to fit the web page’s HTML to parse its content and extract information from it. A common scraping task involves iterating over every possible URL (sometimes called ‘crawling’) and storing data from each page without the risk of human error during extraction. Once the program is complete, all available and desired data can be captured from the web page. This process can be repeated continuously, assuming the web page structure remains mostly the same. (DeVito et al., 2020). Web scraping enables automated identification and extraction of data of interest available on a web page, resulting in scale gain and agility in searching for keywords or text of interest.

For our work, the scraping task will be carried out in PubMed, Science Direct, Ovid Embase, Scientific Electronic Library Online (Scielo), Latin American and Caribbean Health Sciences Literature (Lilacs) databases, and WHO Global Index Medicus between January 2013 to May 2022. The initial year was defined based on the first study describing a target trial emulation (Danaei et al., 2013). The search strategy for scientific articles will be tailored according to the database requirements. Health sciences descriptors (DeCS)/Medical Subject Headings (MeSH) and combined keywords and other indexing terms (i.e., EMTREE), according to the PCC structure, limiting the searches to the concept, will be used. Our interest is to identify what type of publications have mentioned the use of target trial emulations for assessing outcomes in women of reproductive age or the perinatal period exposed to medications. In this regard, we did not limit our search using additional filters for the type of study. The scraping task will be carried out through the fields: title, summary, and keywords. In Scielo, the search will take place using the standard field “All indexes”, which includes the search in the title and abstract of publications. No restrictions on language will be made. Appendix 1 provides the search strategy adopted for this protocol, as well as the description of the data scraping process.

Grey literature will be also retrieved through Web scraping techniques on Google Scholar, which retrieve information from conference proceedings, such as from Epidemiology, Pharmacoepidemiology, and Maternal Health [e.g., Society for Epidemiologic Research [SER] Conference, The Society for Pediatric and Perinatal Epidemiologic Research [SPER] Meeting, Conference on Pharmacoepidemiology and Therapeutic Risk Management, The Canadian National Perinatal Research Meeting (CNPRM)].

Box 1 shows the variables that will be requested at the web scraping stage. Duplicates will be removed through the use of DOI. The automated process will generate an output file with the data provided in an Excel^®^ spreadsheet format to be used in the subsequent phases of this review.

**BOX 1 |:** Variables that will be requested at the web scraping stage.

Variable	Definition
DT	Day/time extraction
TITULO	Title of the article
TIPO_ARTIGO	Type of article - according to the journal classification
PERIODICO	Journal’s name
DIA	Day publication
MES	Month publication
ANO	Year of publication
URL	Uniform Resource Locator
AUTOR_COMPLETO	Complete list of authors
PAIS_AUTOR	Country of the first author
INFO_AUTOR_1	Name first author
DOI	Digital Object Identifier System
DOWNLOAD	website for download of the article
ABSTRACT	Abstract
KEYWORDS	Keywords
BASE	data source

The inclusion criteria will be based on the PCC elements:

#### Population

We will select studies including women of childbearing age, pregnant or postpartum (prior to, during, or after pregnancy). Studies will not be restricted by the range of maternal age. Eligible studies will include primary research studies, systematic reviews, meta-analyses, letters, guidelines, etc.

#### Concept

Emulation of a hypothetical randomised trial using observational data is an approach in which one carefully specifies a causal question and a strategy to minimize the sources of bias in observational research (Hernán and Robins, 2016). However, even if researchers have a high-quality data source, e.g., a database from electronic medical records with millions of patients, emulating a target trial is not a technically trivial exercise, in terms of knowledge, infrastructure or programing skills. Additionally, in pregnancy studies we usually deal with rare outcomes with small numbers of exposed women and consequently limited sample size, as well as relatively short time windows for pregnancy. In this regard, we want to understand what the limitations are in implementing these studies, what questions are being answered, and their respective outcomes in the perinatal period.

#### Context

We want to understand the use of target trial emulation in the preconception, perinatal or postpartum periods. Thus, we will evaluate studies regardless of the data source or country in which they may have been carried out.

Observational studies of medication exposure in the preconception, perinatal and postpartum periods will be eligible for inclusion. We are also planning to assess opinion papers, oral presentations, conference notes, and abstracts. Observational studies comparing pharmacological treatments that have not used a target trial approach will be excluded.

### Stage 3: Study Selection

The authors (LFL and DMM), independently, will select the potentially eligible scientific articles based on the title and abstract, and full text, when necessary, to confirm the relevance of the review question. Through videoconference, the authors will use a sample of two studies to ensure that there was a common understanding of the inclusion and exclusion criteria.

Discrepancies will be discussed and resolved by consensus, by a third reviewer (SMG). The selected studies in this stage will comprise a single database that will be used in the fourth phase of this scoping review. Preferred Reporting Items for Systematic reviews and Meta-Analyses extension for Scoping Reviews (PRISMA-ScR) flow diagram (Tricco et al., 2018) will be followed to reflect the search process.

### Stage 4: Charting the Data

In the single database containing only the eligible studies, we will classify the types of scientific articles according to Lapeña et al. (Lapeña and Peh, 2019): *i)* Primary or original research articles; *ii)* Secondary or review articles; *iii)* Special articles; *iv)* Tertiary literature; and *v)* Grey literature. For articles classified as primary research, we will extract information related to the seven key components of the target trial protocol (Hernán and Robins, 2016). Information to evaluate the characteristics of reporting will be also included. For this, we will use the reporting of studies conducted using observational routinely collected health data statement for pharmacoepidemiology (RECORD-PE) (Langan et al., 2018). Additionally, we will include variables related to the content for perinatal pharmacoepidemiologic research recommended by Margulis et al. (2022). Although evaluating risk of bias in scoping reviews is not currently recommended, we will extract information on characteristics of the target trial emulation, such as treatment strategies being compared (including their start and end times), assignment procedures, follow-up period, outcome, and causal contrast(s) adopted according to Nguyen et al. (2021). The Risk Of Bias In Non-randomised Studies - of Interventions (ROBINS-I) (Sterne et al., 2016) will be used. The variables included represent efforts to identify gaps in the current literature related to the reporting and methods adopted.

### Stage 5: Collating, Summarizing and Reporting the Results

The studies will be grouped according to the variables contained in the single database and characteristics of included studies will synthesized using descriptive statistics, such as absolute numbers and frequencies and measures of central tendency (median) and dispersion (interquartile interval).

### Stage 6: Consultation

We will carry out two consultations. The first consultation will be held at the start of the scoping review to explore experts’ opinions. Researchers experienced with the target trial approach will be consulted on the objectives of our review and asked to provide feedback on whether our aims capture existing knowledge gaps in the field. Researcher’s input may give rise to additional, or modified aims. The findings of the review will be shared with experts (second consultation) to identify any additional gaps not identified in the scoping review. Recommendations for future research will be based on findings of the scoping review and through our consultations with experts.

## Discussion

This protocol presents the background, objectives, and planned methodology for conducting a scoping review to analyze the available literature on the target trial approach for evaluating outcomes in women exposed to medications in the preconception, perinatal or postpartum period.

Data regarding the safety of drugs taken, prior to and during pregnancy and while lactating are lacking and it is necessary to understand how we can answer these questions using rigorous methods in observational research.

We anticipate that, perhaps, few studies will be found, and this may be a possible limitation. However, even if a limited number of studies exist, we will be able to understand to what extent the target trial approach is being used in perinatal pharmacoepidemiology and provide recommendations to improve its use in this field.

## Data Availability

The raw data supporting the conclusion of this article will be made available by the authors, upon request.
